# Economic, cognitive, and social paths of education to health-related behaviors: evidence from a population-based study in Japan

**DOI:** 10.1265/ehpm.22-00178

**Published:** 2023-01-28

**Authors:** Keiko Murakami, Shinichi Kuriyama, Hideki Hashimoto

**Affiliations:** 1 Tohoku Medical Megabank Organization, Tohoku University, 2-1 Seiryo-machi, Aoba-ku, Sendai, Miyagi 980-8573, Japan; 2 Graduate School of Medicine, Tohoku University, 2-1 Seiryo-machi, Aoba-ku, Sendai, Miyagi 980-8575, Japan; 3 Department of Health and Social Behavior, School of Public Health, The University of Tokyo, 7-3-1 Hongo, Bunkyo-ku, Tokyo 113-0033, Japan; 4 Department of Disaster Public Health, International Research Institute of Disaster Science, Tohoku University, 2-1 Seiryo-machi, Aoba-ku, Sendai, Miyagi 980-8573, Japan

**Keywords:** Education, Health literacy, Health-related behaviors, Income, Japan, Social support

## Abstract

**Background:**

There is substantial evidence on the association between lower education and unhealthy behaviors. However, the mechanism underlying this association remains unclear. This study aimed to examine whether income, health literacy, and social support mediate the association between education and health-related behaviors.

**Methods:**

A questionnaire survey was conducted in metropolitan areas in Japan from 2010 to 2011 among residents aged 25–50 years. Data from 3663 participants were used in this study. Health literacy was measured using the Communicative and Critical Health Literacy scale. Health-related behaviors were current smoking, poor dietary habits, hazardous drinking, and lack of exercise. Poisson regression analyses with robust variance estimators were conducted to examine the associations between education and these health-related behaviors. Multiple mediation analyses were conducted to estimate the magnitudes of the mediating effects of income, health literacy, and social support on these associations.

**Results:**

Less educated participants had higher risks of all unhealthy behaviors. Income mediated the associations of education with smoking (6.4%) and exercise (20.0%). Health literacy mediated the associations of education with dietary habits (15.4%) and exercise (16.1%). Social support mediated the associations of education with dietary habits (6.4%) and exercise (7.6%). The education–drinking association was mediated by income in the opposite direction (−10.0%). The proportions of the total effects mediated by income, health literacy, and social support were 9.8% for smoking, 24.0% for dietary habits, −3.0% for drinking, and 43.7% for exercise.

**Conclusions:**

These findings may provide clues for designing effective interventions to reduce educational inequalities in health-related behaviors.

**Supplementary information:**

The online version contains supplementary material available at https://doi.org/10.1265/ehpm.22-00178.

## Background

Health-related behaviors play an important role in shaping population health outcomes for most major diseases worldwide [1]. Health risks can be reduced by adopting healthier behaviors, but many people find it difficult to make lasting changes [2]. Health-related behaviors are influenced by a complex array of interrelated environmental, economic, social, psychological, and biological factors [2]. There is sufficient evidence that socioeconomic status, especially education, is associated with health-related behaviors in developed countries: lower socioeconomic groups more often act in ways that harm their health than higher socioeconomic groups [3].

Diverse mechanisms through which education may influence health-related behaviors have been proposed [3]. One study using a variety of datasets from the United States and the United Kingdom demonstrated that economic resources can account for approximately 20% of the association between education and health-related behaviors, knowledge and cognitive ability explain an additional 40%, and social integration accounts for another 10% [4].

These findings suggest that economic, cognitive, and social mediation paths substantially explain the association between education and health-related behaviors. However, further studies that overcome the following two points would be worthwhile for providing practical implications to close the education-related behavioral gap. First, to the best of our knowledge, no studies, including that conducted by Cutler and Lleras-Muney [4], have examined economic, cognitive, and social mediation paths simultaneously using a single dataset. Therefore, the extents to the different mediation paths independently contribute to behaviors in individuals have not been established. Second, the previous study used a variety of measurement constructs for cognitive ability and social integration with vague conceptual definitions. Indeed, Cutler and Lleras-Muney [4] included social network, social support, and other constructs such as the sense of belongingness adopted in different datasets under the umbrella term of “social integration,” which made it difficult to specify what aspects of the paths affect health-related behaviors. Regarding cognitive ability, the study relied on scores from tests, including arithmetic skills and reading, or intelligence quotient. However, a recent discussion on health literacy indicated that broader cognitive skills to collect, comprehend, and use health-related information beyond functional literacy are required for health promotion [5].

Education is regarded as a major determinant of health literacy [6]. Social support is a key mechanism through which social ties affect health-related behaviors [7]. Therefore, it is reasonable to assume that health literacy and social support mediate the association between education and health-related behaviors. Focusing on these constructs may help us to specify a relevant interventional leverage that can close the education-related health behavioral gap. It has been repeatedly reported that health literacy is associated with health status and health-related behaviors [8]. The studies involved mainly assessed functional health literacy in terms of basic reading and writing skills. Meanwhile, health literacy beyond a basic functional level was also shown to be associated with health status and health-related behaviors in community-dwelling adults in Japan [9, 10], and Japanese adults display the highest levels of proficiency in literacy and numeracy among adults in the OECD countries [11]. We therefore hypothesized that health literacy beyond a basic functional level substantially mediated the associations between education and health-related behaviors in Japan.

Considering the above circumstances, we aimed to determine whether income, health literacy, and social support mediate the association between education and a variety of health-related behaviors simultaneously, using a single dataset from a population-based survey of community-dwelling adults in Japan.

## Methods

### Study design and participants

The sample for the present study was derived from the Japanese Study of Stratification, Health, Income, and Neighborhood (J-SHINE), a population-based survey that was described in detail elsewhere [12–16]. The J-SHINE survey was carried out in four municipalities in and around the greater Tokyo metropolitan area from October 2010 to February 2011. Of 13920 adults aged 25–50 years who were probabilistically selected from the residential registry, survey staff members were able to contact 8408 residents. The participants were asked to complete a computer-assisted and self-administered questionnaire, unless they requested a face-to-face interview. Among them, 4385 residents agreed to participate and complete the survey by providing written informed consent. Valid responses were obtained from 4317 participants, and we analyzed 3663 participants with no missing values for any variables used in the analyses. The characteristics of the 3663 participants who were analyzed and the 654 participants who were excluded are shown in Supplementary Table 1 [see Additional file 1]. The Research Ethics Committee of The University of Tokyo, Graduate School of Medicine approved the survey procedure of the J-SHINE on 28 June, 2010 (No. 3073). The J-SHINE Data Management Committee approved the authors’ secondary use of the data, with personally identifiable information deleted to ensure confidentiality.

### Education

Educational attainment was dichotomized as high school or lower (junior high school, high school) and college or higher (2-year college, special training school, university, graduate school). In Japan at that time, 98.4% of people aged <50 years have completed high school, while 71.6% of high school graduates have completed college or higher education (2010 National Census). Therefore, we chose high school or lower (≤12 years of education) as the cutoff for the educational attainment dummy variable.

### Income, health literacy, and social support

Participants selected their total annual household income from 15 response categories. Equivalent household income was calculated as household income adjusted for household size, using the OECD-modified equivalence scale [17]. For participants whose household income was unknown or missing but who responded on individual income, we used individual income as equivalent household income.

Health literacy was measured using the Communicative and Critical Health Literacy (CCHL) scale [10], which was developed and validated in Japan to assess communicative and critical health literacy [18]. Participants were asked whether they could do the following: collect health-related information from various sources; extract the information they want; understand and communicate the obtained information; consider the credibility of the information; and make decisions based on the information in the context of health issues. Each item was rated on a five-point Likert scale, ranging from *strongly disagree* (1) to *strongly agree* (5). The scores of the five responses were summed and divided by the number of items to determine a total score (theoretical range: 1–5), with higher scores indicating greater health literacy. The Cronbach’s α value of the scale was 0.84.

Social support was measured using the question “How much do the following persons give you helpful guidance when you have a problem or are in trouble?” [13, 19]. In this context, the “following persons” referred to the individual’s spouse/partner, other co-residing family members, non-co-residing family members or relatives, neighbors, and friends. Participants were asked to choose one response option on a five-point Likert scale for each source of support: *a lot* (4), *some* (3), *a little* (2), *never* (1), and *not applicable* (1). The scores for each source of support were summed and divided by the number of items to determine a total score (theoretical range: 1–4), with higher scores indicating greater perceived support.

### Health-related behaviors

Four types of health-related behaviors were measured: current smoking, poor dietary habits, hazardous drinking, and lack of exercise [20]. Participants were dichotomized as current smoker and non-smoker (never smoker, ex-smoker). Dietary habits were measured using five questions (“Do you eat breakfast every day,” “Do you try to eat vegetables,” “Do you try to cut down on sugar and salt intake,” “Do you try to purchase organic vegetables and additive-free food,” and “Do you try to eat nutritionally balanced meals?”) rated on a 5-point scale from *agree* (1) to *disagree* (5). We summed the scores of these five responses to determine a total score (theoretical range, 5–25; Cronbach’s α = 0.75) and defined poor dietary habits as a score of ≥16 [12, 21]. Hazardous drinking was assessed according to average daily alcohol intake calculated from the types and amounts of liquor consumed by the participants. The Health Japan 21 (second term), national health promotion measures from 2013 fiscal year, established targets to reduce the number of people drinking quantities of alcohol that increase the risk of lifestyle-related disease onset: ≥40 g of pure alcohol per day for men and ≥20 g for women [22]. We adopted these national recommendations as the thresholds for hazardous drinking. Habitual exercise was measured by how often the participants engaged in ≥10 minutes of physical activity, excluding incidental exercise related to work, commuting, or other non-leisure behaviors, over the past year. The responses were every day, 5–6 days a week, 3–4 days a week, 1–2 days a week, once a month, or seldom. Lack of exercise was defined as “seldom” [21].

### Statistical analysis

We followed the conventional steps of mediation analyses [23, 24]. We conducted multiple linear regression analyses to examine the association between education and each potential mediator. We also conducted Poisson regression analyses with robust variance estimators to examine the association between education and health-related behaviors after adjustment for age, sex, municipality, marital status (married, unmarried), and work status (working, not working), as well as potential mediators. Prevalence ratios (PRs) and 95% confidence intervals (CIs) were calculated for each health-related behavior.

**Fig. 1:**
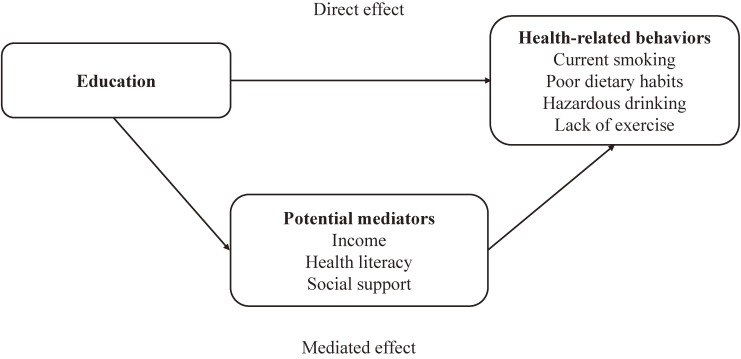
Conceptual framework of the mediation analyses

Based on the results obtained in the above models, we calculated the proportions of the direct effects of education and the effects mediated by each potential mediator using the product of coefficients approach, along with their standard errors, using boot strapping (with 5000 replications). These proportions were used to assess the relative importance and statistical significance of each potential mediator as well as the direct effects of education on health-related behaviors [25]. The conceptual framework of the mediation analyses is illustrated in Fig. 1.

All analyses were conducted using Stata 16.0 (StataCorp LP, College Station, TX, USA). The proportions of the direct and mediated effects were estimated using the *binary_mediation* command in the Stata software.

## Results

Table 1 shows the characteristics of the participants. The percentage of participants with high school education or lower was 22.1%. The percentages of current smoking, poor dietary habits, hazardous drinking, and lack of exercise were 23.6%, 24.3%, 13.6%, and 41.3%, respectively. Lower education was associated with lower levels of equivalent household income, health literacy, and social support [see Additional file 2].

**Table 1:** Characteristics of participants aged 25–50 years residing in metropolitan areas in Japan (n = 3663)

	**mean (SD) or n (%)**	
Age, years, mean (SD)	37.3	(7.2)
Women, n (%)	1924	(52.5)
Municipality, n (%)		
Municipality 1	714	(19.5)
Municipality 2	877	(23.9)
Municipality 3	1081	(29.5)
Municipality 4	991	(27.1)
Married/common-law, n (%)	2585	(70.6)
Working, n (%)	2928	(79.9)
High school education or lower, n (%)	811	(22.1)
Health-related behaviors		
Current smoking, n (%)	863	(23.6)
Poor dietary habits, n (%)	891	(24.3)
Hazardous drinking, n (%)	498	(13.6)
Lack of exercise, n (%)	1512	(41.3)
Possible mediators		
Equivalent household income^a^, mean (SD)	3629.6	(2184.0)
Health literacy, mean (SD) (range 1–5)	3.63	(0.64)
Social support, mean (SD) (range 1–4)	2.36	(0.58)

Abbreviation: SD, standard deviation.

^a^Thousand Japanese yen (/year)

Education was associated with all health-related behaviors after adjustment for age, sex, municipality, marital status, and work status. Specifically, the PRs of high school education or lower compared with college education or higher were 1.80 (95% CI, 1.60–2.02) for current smoking, 1.32 (95% CI, 1.17–1.50) for poor dietary habits, 1.38 (95% CI, 1.15–1.64) for hazardous drinking, and 1.16 (95% CI, 1.06–1.27) for lack of exercise [see Additional file 3].

Table 2 shows the association of education, income, health literacy, and social support with health-related behaviors, after adjustment for age, sex, municipality, marital status, work status, and education/income/health literacy/social support. The association with current smoking and the association with poor dietary habits were attenuated but did not disappear; the PRs of high school education or lower compared with college education or higher were 1.70 (95% CI, 1.52–1.91) and 1.25 (95% CI, 1.11–1.42), respectively. Lower income was associated with current smoking, while health literacy and social support were not. Lower health literacy and less social support were associated with poor dietary habits, while income was not. The association of education with hazardous drinking was amplified; the corresponding PR was 1.41 (95% CI, 1.18–1.68). Higher income was associated with hazardous drinking, while health literacy was not. The association of education with lack of exercise was nullified; the corresponding PR was 1.09 (95% CI, 0.99–1.19). Lower income, lower health literacy, and less social support were associated with lack of exercise.

**Table 2:** Associations of education, income, health literacy, and social support with health-related behaviors

	**Current smoking**		**Poor dietary habits**		**Hazardous drinking**		**Lack of exercise**	
	**PR (95% CI)**		**PR (95% CI)**		**PR (95% CI)**		**PR (95% CI)**	
Educational attainment								
College or higher	1.00		1.00		1.00		1.00	
High school or lower	1.70	(1.52, 1.91)	1.25	(1.11, 1.42)	1.41	(1.18, 1.68)	1.09	(0.99, 1.19)
Equivalent household income								
4th quartile	1.00		1.00		1.00		1.00	
3rd quartile	1.06	(0.89, 1.27)	1.09	(0.92, 1.30)	1.02	(0.82, 1.27)	1.02	(0.90, 1.17)
2nd quartile	1.22	(1.03, 1.44)	1.15	(0.98, 1.35)	0.82	(0.65, 1.03)	1.26	(1.12, 1.41)
1st quartile	1.37	(1.15, 1.62)	1.06	(0.89, 1.26)	0.82	(0.64, 1.05)	1.29	(1.14, 1.45)
*p* for trend	<0.001		0.44		0.038		<0.001	
Health literacy								
4th quartile	1.00		1.00		1.00		1.00	
3rd quartile	1.10	(0.91, 1.33)	1.12	(0.91, 1.40)	0.93	(0.72, 1.19)	1.25	(1.08, 1.44)
2nd quartile	1.10	(0.93, 1.31)	1.42	(1.18, 1.71)	0.86	(0.68, 1.08)	1.28	(1.12, 1.46)
1st quartile	1.16	(0.98, 1.38)	1.74	(1.45, 2.10)	1.04	(0.82, 1.33)	1.47	(1.29, 1.68)
*p* for trend	0.11		<0.001		0.85		<0.001	
Social support								
4th quartile	1.00		1.00		1.00		1.00	
3rd quartile	0.97	(0.80, 1.18)	1.19	(0.94, 1.49)	1.07	(0.81, 1.40)	1.04	(0.92, 1.17)
2nd quartile	0.96	(0.80, 1.16)	1.43	(1.15, 1.78)	1.23	(0.95, 1.60)	1.10	(0.97, 1.24)
1st quartile	1.08	(0.90, 1.29)	1.65	(1.33, 2.04)	1.29	(0.99, 1.68)	1.29	(1.15, 1.46)
*p* for trend	0.29		<0.001		0.035		<0.001	

Abbreviation: PR, prevalence ratio; 95% CI, 95% confidence interval.

PR represents the risk of health-related behaviors in the 1st, 2nd, and 3rd quartile groups compared with the 4th quartile group.

Adjusted for age, sex, municipality, marital status, work status, and educational attainment/equivalent household income/health literacy/social support.

Equivalent household income: 1st, 2nd, 3rd, and 4th quartiles correspond to ≤2250, 2251–3473, 3474–4500, and ≥4501 thousand Japanese yen, respectively.

Health literacy: 1st, 2nd, 3rd, and 4th quartiles correspond to ≤3.2, 3.3–3.8, 3.9–4.0, and ≥4.1, respectively.

Social support: 1st, 2nd, 3rd, and 4th quartiles correspond to ≤2.0, 2.1–2.4, 2.5–2.8, and ≥2.9, respectively.

Table 3 presents the proportions of the direct and mediated effects of income, health literacy, and social support on the association between education and health-related behaviors. Income partially mediated the associations of education with current smoking (6.4%) and lack of exercise (20.0%), but partially mediated the association with hazardous drinking in the opposite direction (−10.0%). Health literacy partially mediated the associations of education with poor dietary habits (15.4%) and lack of exercise (16.1%). Social support partially mediated the associations of education with poor dietary habits (6.4%) and lack of exercise (7.6%). The proportions of the total effects mediated by income, health literacy, and social support were 9.8% for current smoking, 24.0% for poor dietary habits, −3.0% for hazardous drinking, and 43.7% for lack of exercise.

**Table 3:** Proportions of the direct and indirect effects on the association between education and health-related behaviors

	**Current smoking**		**Poor dietary habits**		**Hazardous drinking**		**Lack of exercise**	
	**%**	**(95% CI)**	**%**	**(95% CI)**	**%**	**(95% CI)**	**%**	**(95% CI)**
Direct effect	90.2	(84.9, 94.6)	76.0	(53.4, 87.7)	103.0	(91.3, 117.5)	56.3	(−16.8, 75.5)
Mediated effect via:								
Equivalent household income	6.4	(2.8, 11.3)	2.2	(−6.2, 10.8)	−10.0	(−29.6, −1.3)	20.0	(8.6, 58.7)
Health literacy	2.4	(0.4, 5.0)	15.4	(8.5, 31.0)	4.0	(−1.3, 14.5)	16.1	(7.4, 41.6)
Social support	1.0	(0.02, 2.8)	6.4	(2.1, 14.8)	3.0	(0.2, 12.1)	7.6	(2.4, 23.5)
Total mediated effect	9.8	(5.4, 15.1)	24.0	(12.3, 46.6)	−3.0	(−17.5, 8.7)	43.7	(24.5, 116.8)
Total	100.0		100.0		100.0		100.0	

Abbreviation: 95% CI, 95% confidence interval.

The percentages represent the extent to which the variables explain the association between education and health-related behaviors.

Adjusted for age, sex, municipality, marital status, and work status.

## Discussion

Participants with lower education had higher risks of unhealthy behaviors (Table 2). Lower income mediated the associations of lower education with current smoking and lack of exercise (Table 3). Lower health literacy mediated the associations of lower education with poor dietary habits and lack of exercise (Table 3). Less social support mediated the associations of lower education with poor dietary habits and lack of exercise (Table 3). The proportions of the total effects mediated by these factors varied substantially across the targeted behaviors.

The proportions of the effects mediated by income were 6.4% for smoking and 20.0% for lack of exercise. Theoretically, education is a precursor for higher income through human capital formation, and higher income would lead to larger economic resources that enable healthier behavioral choices. Our results indicated that this theory applied to exercise, but not to smoking. One systematic review also concluded that availability of physical activity equipment and accessibility and convenience of recreational facilities were associated with physical activity/sports [26]. Another possible mechanism may be related to time poverty [27]. Although high-income earners would have time poverty, their high income may allow them to buy resources and opportunities that permit efficient use of time for physical exercise. These results are consistent with our finding of income mediation for the education–exercise association. We also found an association between lower income and current smoking, consistent with the finding in a previous meta-analysis that lower income was consistently associated with high smoking prevalence [28]. A study in Japan using data from a national survey on household consumption indicated that households with higher income spent more money on education than on tobacco compared with households with lower income [29]. Thus, income may not be a mediator, but rather a magnifier that enlarges the education-related gap in smoking.

The proportions of the effects mediated by health literacy were 2.4% for smoking, 15.4% for poor dietary habits and 16.1% for lack of exercise. The small mediation by health literacy for the education–smoking association may be explained by evidence that knowledge of the risks of smoking is widespread and does little to account for socioeconomic inequalities in smoking [3]. The mediation by health literacy for the associations of education with dietary habits and exercise was substantial. A study in Denmark showed that health literacy mediated the associations of education with diet and physical activity [30]. Another study in the United States showed that health literacy mediated the associations of education with intake of fruits and vegetables and physical activity [31]. Our results are consistent with these previous studies. A systematic review concluded that food literacy, as a specific form of health literacy, can significantly contribute to guidance of future health promotion activities focusing on dietary behavior [32]. However, health literacy has attracted little attention in the context of physical activity/exercise [33]. We found that lower health literacy was associated with not only poor dietary habits but also lack of exercise. Our results indicate that more attention should be paid to health literacy when considering the associations of education with dietary habits and exercise.

The proportions of the effects mediated by social support were 6.4% for poor dietary habits and 7.6% for lack of exercise. Although previous findings indicate the important role of social support in dietary habits and physical activity [26, 34], the mediating role of social support for the education–behavior association appears inconsistent [31, 35]. There are two possible explanations for the small mediating effect in the present study. One is that the measure of social support in the present study was quite generic, rather than explicitly tied to having support for health-related behaviors. The second is that other types of measures of social relationships may have more mediating effects than social support, because different measures of social relationships seem to be differently associated with health-related behaviors [7]. The proportion of the effects mediated by social support was 1.0% for smoking. A previous study on the J-SHINE survey demonstrated that popular peers’ smoking was differently associated with ego’s smoking depending on ego’s educational attainment [16]. This finding suggests that the social network, rather than social support, explains the association between education and smoking.

The present findings have implications for the design of effective interventions aiming to reduce educational inequalities in health behaviors. Income, health literacy, and social support have different mediating effects on the associations between education and different behaviors. These results suggest that different intervention strategies are needed to reduce educational inequalities in different behaviors. Furthermore, health literacy substantially mediated the associations of education with dietary habits and exercise. Relatively few interventions to improve health literacy conducted in community settings have incorporated the concepts of critical health literacy [36, 37]. The CCHL scale used in the present study explicitly attempts to assess critical health literacy in terms of information appraisal by asking respondents the extent to which they consider the reliability, validity, credibility, and applicability of health information [10, 38]. A previous study showed that health literacy measured by the CCHL scale was significantly improved after an educational program that provided participants with the necessary knowledge and skills in a community setting [39]. Our findings suggest that educational inequalities in health-related behaviors would be reduced by interventions that actively use the concept of health literacy, especially critical health literacy, in their design and evaluation.

To the best of our knowledge, the present study is the first to simultaneously examine economic, cognitive, and social mediation paths of education to health-related behaviors using a single dataset, which is the strength of the study. However, limitations of the present study should be noted. First, our findings are based on cross-sectional data, and therefore no conclusions about temporality and causation can be made. Second, the J-SHINE survey had a relatively low response rate, although the respondents were fairly comparable with the target population in age, sex, and educational attainment [15]. Approximately 15% of the participants who provided valid responses were excluded from the analyses due to missing values for any variables used in the analyses. The participants excluded from the analyses were more likely to have lower education, although no differences in the prevalences of health-related behaviors except current smoking were found between the participants who were analyzed and those who were not [see Additional file 1]. These factors may have led to an underestimation of the association between education and health-related behaviors. Third, the sampled municipalities were all located in urban areas. Our findings should only be generalized with caution, because neighborhood environments were demonstrated to be associated with dietary habits and physical activity [40, 41]. In addition, our findings do not necessarily apply to other countries, because different countries have different educational levels. Fourth, the variables were all self-reported, and may have been subject to influence by social desirability bias. Although the questions and definitions of poor dietary habits and lack of exercise in the present study were also used in previous studies [12, 21], validated questionnaires would be useful for more precise assessments. Fifth, the present study only used exercise as a measure of physical activity, although different factors were shown to be associated with different types of physical activity [26]. Nevertheless, leisure-time activities contribute more to total physical activity in high-income countries than elsewhere [42]. Finally, the direct effects of education remain substantial, especially for current smoking and hazardous drinking. Other potential mediators should be examined to clarify the detailed mechanism through which education influences health-related behaviors.

## Conclusions

The present study clarified that different health-related behaviors were mediated by different path components. This finding suggests that different intervention strategies are needed to reduce educational inequalities in different behaviors. It may also provide clues for designing effective interventions to reduce educational inequalities in health-related behaviors such as interventions that actively use the concept of health literacy in their design and evaluation, because targeting evidence-based mediators in interventions is a crucial step toward improvement of the effectiveness of these interventions.

## Abbreviations

CCHL: communicative and critical health literacy
J-SHINE: Japanese Study of Stratification, Health, Income, and Neighborhood
PR: prevalence ratio
95% CI: 95% confidence interval

## Supplementary information

**Additional file 1:** **Supplementary Table 1.** Characteristic differences between 3663 participants who were analyzed and 654 participants who were excluded from the analysis.

**Additional file 2:** **Supplementary Table 2.** Associations of education with income, health literacy, and social support.

**Additional file 3:** **Supplementary Table 3.** Associations of education, income, health literacy, and social support with health-related behaviors.

## Declarations

### Availability of data and material

The use of J-SHINE data is to be approved by the J-SHINE Data Management Committee by request.
