# Supplementary material for: Economic, cognitive, and social paths of education to health-related behaviors: evidence from a population-based study in Japan
Source: Environ Health Prev Med. 2023 Jan 28;28:9. doi: 10.1265/ehpm.22-00178 (PMC9884565; doi:10.1265/ehpm.22-00178)
Supplement: Supplementary file 1 — Additional file 1: Supplementary Table 1. Characteristic differences between 3663 participants who were analyzed and 654 participants who were excluded from the analysis. [file ehpm-28-009-s001.docx]

**Additional file 1**

| **Supplementary Table 1. Characteristic differences between 3663 participants who were analyzed and 654 participants who were excluded from the analysis** | | | | | |
| --- | --- | --- | --- | --- | --- |
|  | Participants who were analyzed (n=3663) | | Participants who were not analyzed (n=654) | | *p*-value^a^ |
| Age, years, mean (SD) | 37.3 | (7.2) | 37.3 | (7.6) | 0.84 |
| Women, n (%) | 1924 | (52.5) | 389 | (59.5) | 0.001 |
| Municipality, n (%) |  |  |  |  | 0.46 |
| Municipality 1 | 714 | (19.5) | 134 | (20.5) |  |
| Municipality 2 | 877 | (23.9) | 153 | (23.4) |  |
| Municipality 3 | 1081 | (29.5) | 176 | (26.9) |  |
| Municipality 4 | 991 | (27.1) | 191 | (29.2) |  |
| Married/common-law, n (%) | 2585 | (70.6) | 404 | (62.2) | <0.001 |
| Working, n (%) | 2928 | (79.9) | 465 | (72.2) | <0.001 |
| High school education or lower, n (%) | 811 | (22.1) | 188 | (31.8) | <0.001 |
| Health-related behaviors |  |  |  |  |  |
| Current smoking, n (%) | 863 | (23.6) | 183 | (28.2) | 0.010 |
| Poor dietary habits, n (%) | 891 | (24.3) | 167 | (25.6) | 0.48 |
| Hazardous drinking, n (%) | 498 | (13.6) | 68 | (12.8) | 0.62 |
| Lack of exercise, n (%) | 1512 | (41.3) | 261 | (40.6) | 0.74 |
| Possible mediators |  |  |  |  |  |
| Equivalent household income^b^, mean (SD) | 3629.6 | (2184.0) | 3400.0 | (2232.7) | 0.17 |
| Health literacy, mean (SD) (range 1–5) | 3.63 | (0.64) | 3.57 | (0.67) | 0.013 |
| Social support, mean (SD) (range 1–4) | 2.36 | (0.58) | 2.33 | (0.66) | 0.23 |
| Abbreviation: SD, standard deviation. | | | | | |
| Percentages are shown after excluding those whose values were missing. | | | | | |
| ^a^Obtained using the Student’s *t*-test for continuous variables and the chi-squared test for categorical variables, comparing participants who were analyzed and those who were not. | | | | | |
| ^b^Thousand Japanese yen (/year) | | | | | |
