# Supplementary material for: Economic, cognitive, and social paths of education to health-related behaviors: evidence from a population-based study in Japan
Source: Environ Health Prev Med. 2023 Jan 28;28:9. doi: 10.1265/ehpm.22-00178 (PMC9884565; doi:10.1265/ehpm.22-00178)
Supplement: Supplementary file 2 — Additional file 2: Supplementary Table 2. Associations of education with income, health literacy, and social support. [file ehpm-28-009-s002.docx]

**Additional file 2**

| **Supplementary Table 2. Associations of education with income, health literacy, and social support** | | | | | | | | | |
| --- | --- | --- | --- | --- | --- | --- | --- | --- | --- |
|  |  | Equivalent household income^a^ | |  | Health literacy | |  | Social support | |
|  |  | Coefficient (95% CI) | |  | Coefficient (95% CI) | |  | Coefficient (95% CI) | |
| Educational attainment |  |  |  |  |  |  |  |  |  |
| College or higher |  | Reference |  |  | Reference |  |  | Reference |  |
| High school or lower |  | –797.26 | (–963.41, –631.12) |  | –0.14 | (–0.19, –0.09) |  | –0.06 | (–0.10, –0.02) |
| Abbreviation: 95% CI, 95% confidence interval. | | | | | | | | | |
| The coefficients represent the equivalent household income, health literacy, and social support in the high school or lower education group compared with the college or higher education group. | | | | | | | | | |
| Adjusted for age, sex, municipality, marital status, and work status. | | | | | | | | | |
| ^a^Thousand Japanese yen (/year) | | | | | | | | | |
