# Supplementary material for: Economic, cognitive, and social paths of education to health-related behaviors: evidence from a population-based study in Japan
Source: Environ Health Prev Med. 2023 Jan 28;28:9. doi: 10.1265/ehpm.22-00178 (PMC9884565; doi:10.1265/ehpm.22-00178)
Supplement: Supplementary file 3 — Additional file 3: Supplementary Table 3. Associations of education, income, health literacy, and social support with health-related behaviors. [file ehpm-28-009-s003.docx]

**Additional file 3**

| **Supplementary Table 3. Associations of education, income, health literacy, and social support with health-related behaviors** | | | | | | | | |
| --- | --- | --- | --- | --- | --- | --- | --- | --- |
|  | Current smoking | | Poor dietary habits | | Hazardous drinking | | Lack of exercise | |
|  | PR (95% CI) | | PR (95% CI) | | PR (95% CI) | | PR (95% CI) | |
| Educational attainment |  |  |  |  |  |  |  |  |
| College or higher | 1.00 |  | 1.00 |  | 1.00 |  | 1.00 |  |
| High school or lower | 1.80 | (1.60, 2.02) | 1.32 | (1.17, 1.50) | 1.38 | (1.15, 1.64) | 1.16 | (1.06, 1.27) |
| Equivalent household income |  |  |  |  |  |  |  |  |
| 4th quartile | 1.00 |  | 1.00 |  | 1.00 |  | 1.00 |  |
| 3rd quartile | 1.11 | (0.93, 1.33) | 1.16 | (0.98, 1.38) | 1.05 | (0.85, 1.30) | 1.05 | (0.92, 1.19) |
| 2nd quartile | 1.33 | (1.12, 1.56) | 1.28 | (1.09, 1.50) | 0.86 | (0.69, 1.08) | 1.32 | (1.17, 1.48) |
| 1st quartile | 1.55 | (1.31, 1.84) | 1.20 | (1.01, 1.43) | 0.90 | (0.71, 1.15) | 1.37 | (1.22, 1.54) |
| *p* for trend | <0.001 | | 0.018 | | 0.18 | | <0.001 | |
| Health literacy |  |  |  |  |  |  |  |  |
| 4th quartile | 1.00 |  | 1.00 |  | 1.00 |  | 1.00 |  |
| 3rd quartile | 1.11 | (0.91, 1.34) | 1.14 | (0.92, 1.41) | 0.94 | (0.73, 1.20) | 1.25 | (1.08, 1.44) |
| 2nd quartile | 1.16 | (0.98, 1.38) | 1.47 | (1.22, 1.77) | 0.88 | (0.69, 1.10) | 1.33 | (1.16, 1.51) |
| 1st quartile | 1.29 | (1.08, 1.54) | 1.84 | (1.53, 2.22) | 1.06 | (0.84, 1.35) | 1.56 | (1.37, 1.78) |
| *p* for trend | 0.003 | | <0.001 | | 0.73 | | <0.001 | |
| Social support |  |  |  |  |  |  |  |  |
| 4th quartile | 1.00 |  | 1.00 |  | 1.00 |  | 1.00 |  |
| 3rd quartile | 0.96 | (0.79, 1.17) | 1.17 | (0.93, 1.47) | 1.07 | (0.81, 1.40) | 1.03 | (0.91, 1.17) |
| 2nd quartile | 0.95 | (0.78, 1.15) | 1.43 | (1.15, 1.78) | 1.22 | (0.94, 1.58) | 1.10 | (0.98, 1.25) |
| 1st quartile | 1.11 | (0.92, 1.32) | 1.71 | (1.38, 2.12) | 1.28 | (0.98, 1.68) | 1.33 | (1.18, 1.50) |
| *p* for trend | 0.17 | | <0.001 | | 0.038 | | <0.001 | |
| Abbreviation: PR, prevalence ratio; 95% CI, 95% confidence interval. | | | | | | | | |
| PR represents the risk of health-related behaviors in the 1st, 2nd, and 3rd quartile groups compared with the 4th quartile group. | | | | | | | | |
| Adjusted for age, sex, municipality, marital status, and work status. | | | | | | | | |
| Equivalent household income: 1st, 2nd, 3rd, and 4th quartiles correspond to ≤2250, 2251–3473, 3474–4500, and ≥4501 thousand Japanese yen, respectively. | | | | | | | | |
| Health literacy: 1st, 2nd, 3rd, and 4th quartiles correspond to ≤3.2, 3.3–3.8, 3.9–4.0, and ≥4.1, respectively. | | | | | | | | |
| Social support: 1st, 2nd, 3rd, and 4th quartiles correspond to ≤2.0, 2.1–2.4, 2.5–2.8, and ≥2.9, respectively. | | | | | | | | |
